# Floral Species Richness Correlates with Changes in the Nutritional Quality of Larval Diets in a Stingless Bee

**DOI:** 10.3390/insects11020125

**Published:** 2020-02-15

**Authors:** Moritz Trinkl, Benjamin F. Kaluza, Helen Wallace, Tim A. Heard, Alexander Keller, Sara D. Leonhardt

**Affiliations:** 1 Department of Animal Ecology and Tropical Biology, University of Würzburg, 97074 Würzburg, Germany; moritz_trinkl@web.de; 2 Department of Public Technology and Innovation Planning, Fraunhofer Institute for Technological Trend Analysis INT, 53879 Euskirchen, Germany; 3 Environmental Futures Research Institute, Griffith University, Nathan Campus, QLD 4111, Australia; helen.wallace@griffith.edu.au; 4 CSIRO Ecosystem Sciences, Brisbane, QLD 4001, Australia; tim@sugarbag.net; 5 Center for Computational and Theoretical Biology, University of Würzburg, 97074 Würzburg, Germany; a.keller@biozentrum.uni-wuerzburg.de; 6 Department of Ecology and Ecosystem Management, Technical University of Munich, 85354 Freising, Germany

**Keywords:** floral resources, plant-insect interactions, nutrition, biodiversity, bee decline

## Abstract

Bees need food of appropriate nutritional quality to maintain their metabolic functions. They largely obtain all required nutrients from floral resources, i.e., pollen and nectar. However, the diversity, composition and nutritional quality of floral resources varies with the surrounding environment and can be strongly altered in human-impacted habitats. We investigated whether differences in plant species richness as found in the surrounding environment correlated with variation in the floral diversity and nutritional quality of larval provisions (i.e., mixtures of pollen, nectar and salivary secretions) composed by the mass-provisioning stingless bee *Tetragonula carbonaria* (Apidae: Meliponini). We found that the floral diversity of larval provisions increased with increasing plant species richness. The sucrose and fat (total fatty acid) content and the proportion and concentration of the omega-6 fatty acid linoleic acid decreased, whereas the proportion of the omega-3 fatty acid linolenic acid increased with increasing plant species richness. Protein (total amino acid) content and amino acid composition did not change. The protein to fat (P:F) ratio, known to affect bee foraging, increased on average by more than 40% from plantations to forests and gardens, while the omega-6:3 ratio, known to negatively affect cognitive performance, decreased with increasing plant species richness. Our results suggest that plant species richness may support *T. carbonaria* colonies by providing not only a continuous resource supply (as shown in a previous study), but also floral resources of high nutritional quality.

## 1. Introduction

Pollinators, like all other animals, need food of appropriate amounts and quality to survive and reproduce. Some pollinators, in particular bees, obtain all required nutrients from flowering plants through collecting pollen and/or nectar as ‘reward’ for their ‘pollination service’. Nectar mostly provides sugars, while pollen supplies most macro- and micronutrients, i.e., protein/amino acids, carbohydrates, lipids/fatty acids, vitamins, sterols and trace elements [1,2,3].

In both solitary and social bee species, females construct cells, each of which is mass provisioned with a mixture of pollen and nectar, occasionally enriched with glandular secretions, and one egg [4]. The larva emerges and normally only feeds on the previously provisioned food, except for honeybees (*Apis*), some bumblebee (*Bombus*) species and many Allodapini species where larvae are fed progressively by adult bees [4]. Unfortunately, we still know very little about the precise nutritional requirements for proper larval development in most bee species. Information is largely restricted to *Apis mellifera* [2]. Besides pollen, honeybee larvae are fed with jelly, which contains on average 14% protein, 6% lipids, 18% carbohydrates (wet weight), a protein to carbohydrate ratio (P:C) of 1:1 and a protein to lipids ratio (P:L) of 2.2:1, with the lipid content being most variable [2]. If bees have less access to pollen and, consequently, the nutrients it provides, colonies cease larval production or produce larvae with malformations and other impairments due to reduced food quantity and/or quality [5].

Social bees typically are generalist flower visitors [6]. They collect pollen and nectar from a comparatively large spectrum of different plant species [6] but are generally more selective in their choice of pollen than of nectar plants [7,8]. The floral composition of collected pollen (and likely also nectar) is strongly affected by the composition of plant communities available in the surrounding environment [9,10]. It directly affects the health, performance and fitness of bee colonies, with health and fitness often increasing with pollen floral diversity [11,12,13,14,15] and depending on floral composition [15].

Both species diversity and composition of plant communities visited by bees for pollen and nectar collection are strongly affected by anthropogenic activities [16,17,18]. Such activities often convert natural habitats into impoverished landscapes with reduced floral and thus resource availability and diversity [19,20,21,22,23]. Reduced floral diversity is typically linked to reduced plant biomass and productivity and negatively affects the abundance and diversity of primary consumers, including bees [24,25,26,27,28,29,30]. It may be one significant driver of bee declines [20,31,32]. Two studies investigated how the surrounding foraging landscape and thus floral resource diversity and composition affected the nutritional quality of bee bread allocated by *Apis mellifera* colonies in Great Britain [10,33]. Both the floral composition and the nutritional quality of bee bread were strongly affected by the surrounding foraging landscape: bee bread of higher protein content was found in environments that were comparatively less affected by anthropogenic activities [10,33]. Two studies on bumblebees did not find an effect of landscape on variation in the protein content and protein-to-lipid ratio of allocated pollen [9,34].

In eusocial bees, such as *Apis* honeybees and stingless bees (Apidae: Meliponini), part of the allocated pollen (or bee bread) is mixed with nectar/honey and glandular secretions and fed to the colony’s offspring either progressively (as in *Apis mellifera*) or through mass provisioning (as in stingless bees) reviewed by [2]. Honeybee larvae are also fed with (pure) pollen at later larval stages [2]. In contrast, stingless bee larvae are raised entirely on a semi-liquid diet mixed from pollen and nectar collected over prolonged periods, consisting mostly of water (40–60% of wet mass) and sugar (5–12%) [35]. In stingless bees, these larval provisions should, therefore, be of appropriate nutritional quality to best support larval development. Adults cannot respond to signals of growing larvae and potentially adjust their provisions, because cells are capped once they are provisioned. This may also explain why, for some stingless bee species, the nutritional composition of larval provisions is similar between different colonies [35]. The nutritional quality of larval provisions may be affected by the composition of floral resources, i.e., pollen and nectar, used for provisioning. However, within-nest processing, such as pollen storage and the addition of glandular secretions, may also alter nutritional quality [2]. Nutritional analyses of bee larval provisions in relation to available floral diversity are lacking. Better understanding the link between nutritional quality of larval provisions and floral resource diversity/abundance in the surrounding environment would give potential new insight into the role of available floral resource diversity and composition for bee health. It would also show if the diversity of available floral resources affects the quantity or quality of allocated resources (or both) and, subsequently, pollinator fitness and population dynamics.

We used the Australian stingless bee *Tetragonula carbonaria* to investigate how plant species richness and resource abundance in the surrounding environment affected the floral diversity and nutritional quality of larval provisions in a mass provisioning bee. We hypothesized that the floral richness of larval provisions would increase with increasing plant species richness in the surrounding environment since *T. carbonaria* foragers collected more diverse pollen sources in more diverse environments [36]. We further hypothesized that at least some pollen nutritional quality parameters would change with increasing surrounding plant species richness and/or floral diversity in larval provisions. Previous studies showed that bumblebees preferentially foraged on pollen with high P:F ratios [37,38] and that honeybees balanced fatty acid ratios [39,40], indicating that the protein and/or fat content of pollen might affect foraging choices across Apidae species. We, therefore, predicted that the protein to fat (P:F) ratio would increase with increasing floral diversity.

## 2. Material and Methods

### 2.1. Study Sites

The study was conducted in Queensland, Australia, with the sample collection taking place between 2011 and 2016. The east coast of Queensland is characterized by a subtropical climate with wet summer and dry winter seasons. The landscape is heterogeneous with intermingled agricultural, urban and natural areas and harbors a very species diverse vegetation (>14,000 plant species) [41,42].

To test if the floral diversity of the surrounding environment affects the floral and nutritional composition of larval provisions, we collected larval provisions from colonies located along a gradient of increasing (surrounding) plant species richness. This gradient comprised three different habitat types, i.e., agricultural plantations (21 samples from 16 colonies), natural forests (31 samples from 16 colonies) and urban gardens (36 samples from 29 colonies). We included sites from Brisbane in the south (27°29″ S, 153°1″ E) to Bundaberg (26°43″ S, 153°1″ E) in the north of Queensland (maximum distance between sites >300 km) [36,43,44] (Table S1). All sites were at about sea level. Plant species richness surrounding colonies was assessed through botanical surveys along four 500 m transects in four directions (north, south, east and west, each 5 m wide), conducted once for each study site between 2012 and 2014. We chose 500 m because this is the maximum flight radius of *Tetragonula carbonaria* [45]. All plant species within a 5 m wide corridor along these 1000 m long transects were identified. Observations of flower visits in the field indicate that *T. carbonaria* visits a large spectrum of different plant species, including many eucalypts, *Leptospermum* and other Myrtaceae, *Banksia*, *Macadamia* and other Proteaceae, as well as many herb species, etc. (Wallace HM, Leonhardt SD, Heard TA, personal observations). However, there is no information available on all resource plants potentially used by Australian stingless bees, which is why we considered all flowering plants to provide some sort of resource, either pollen or nectar, while we excluded plants that did not provide any floral resources (i.e., ferns and grasses). Our natural forest sites comprised relatively open heathland, dominated by the plant genus *Banksia* (Proteaceae), or denser sclerophyll forests with a closed canopy dominated by eucalypt species. Plantation sites comprised commercial macadamia plantations (*Macadamia integrifolia* Maiden and Betche X *M. tetraphylla* Johnson), and urban gardens typically comprised gardens of 300–1000 m^2^ with both native and exotic plants. Plant species richness significantly increased from plantations to urban gardens [36]. We further quantified the proportional area of each habitat type (plantation, forest, garden) for each site within the flight radius of *Tetragonula carbonaria*. All habitats within a 500 m radius were classified using aerial photos obtained by Google Earth (software: KML Toolbox, Zonum Solutions, 2012). On average, 90% of the area was covered by plantations at sites classified as “plantation” (with the remaining habitat being forest), 90% of the area was covered by forest at “forest” sites (with the remaining area being fields and gardens), and 82% of “garden” sites were covered by gardens (with the remainder being water). The transects used for botanical surveys always covered mostly the target habitat. Habitat patches identified for each site were validated by further ground surveys independent of transect walks [43].

### 2.2. Study Species

We used colonies of the Australian stingless bee *T. carbonaria* (Apidae, Meliponini, genus change: [46,47]). This species is native to the study region, but can also be kept and propagated in hive boxes (Figure S1) [48]. It lives in long-lived colonies with up to several thousand workers and one single-mated queen [48]. Like most stingless bee species [49], it forages on a broad spectrum of different plant species from different families, with clear preferences for some species over others (Wallace HM, Leonhardt SD, Heard TA, personal observations), but the precise spectrum of plant species visited by *T. carbonaria* for resource allocation has not yet been determined. Colonies of *T. carbonaria* were kept in hives (consisting of two boxes housing the brood and an additional box used as honey super, Figure S1) and artificially propagated to extract samples and measure their reproductive output following Heard [48].

In 2011, we placed four *T. carbonaria* colonies (in hives) at each of four sites (replicates) per habitat type with a minimum distance of 1.1 km in between two sites (i.e., minimum distances between sites were 1.1 km in plantations, 14.3 km in forests and 1.4 km in gardens), resulting in 12 sites, which were used throughout the entire study period. Two original colonies had to be excluded as they were usurped by another (non-target) stingless bee species. As a result, 46 original *T. carbonaria* bee hives were used at the beginning of the study. These colonies reproduced at different rates, resulting in a total of 93 bee hives by March 2014 (mean ± standard deviation; plantations: 3 ± 2 per site; forests: 3 ± 2; gardens: 6 ± 4) [44].

### 2.3. Sampling of Larval Provisions

*Tetragonula carbonaria* has a circular brood in the shape of an upright elongated sphere (ellipsoid), which is surrounded by honey and pollen storage pots (Figure S1). Brood cells are mass provisioned and then sealed. The brood forms a spiral that continuously grows upwards when new brood cells are built [50,51] (Figure S1). The part of the spiral with cell construction and provisioning is referred to as advancing front (Figure S1) and can strongly differ in size depending on the colony’s overall provisioning stage. Each hive harboring one bee colony was opened once per year in 2011, 2012 and 2013 (plus January 2014), and then again in 2016, to record colony performance and to obtain larval provision samples. The order of colony openings followed a protocol that ensured that at least one colony was opened at each site in each season (wet, cold and dry) (see Table S1 for specific dates). When a hive was opened, brood and food storage were separated by a horizontal cut between the center and bottom box, aiming at exposing the advancing front (Figure S1). However, due to differences in the colonies’ developmental stages, brood sizes and brood structures, the advancing front could not always be exposed through cutting, which prevented the sampling of larval provisions. In our study, we hit the advancing front in 36% of sampled colonies, which resulted in an unbalanced final data set (e.g., some colonies were sampled multiple times across years, others just once, see Table S1).

Where possible, we collected between 3 and 20 (mean ± SD: 7 ± 3) freshly capped neighboring brood cells per colony/hive. The amount of brood cells collected represented approximately 10–20% of recently provisioned brood cells and depended on the colony’s brood size [48]. If feasible, we sampled each colony multiple times over the five year period (mean sampling number 1.5 ± 0.8, minimum sampling number: 1, maximum sampling number: 4). Because *T. carbonaria* consecutively provision brood cells, neighboring brood cells of the advancing front were most likely provisioned and capped around the same time and were thus of about equal age. Brood cells were opened in the laboratory and larval provisions were extracted using a micropipette. The provisions of 3–7 cells (depending on the size of the colony/brood and thus brood cells available) were combined in one Eppendorf tube and weighed to obtain the average (wet) amount of larval provision [in mg ± standard variation] per cell (6.92 ± 1.12), resulting in 88 samples from 61 colonies overall (1–4 samples per colony, Table S1). Each sample contained several brood cells and each brood cell contained a mix of pollen, nectar and salivary secretions (composed from several storage and thus longer time periods pots by nurses) [48]. Moreover, as many colonies were sampled several times, we were confident that the number of samples obtained enabled a robust analysis. Variation in the amounts of larval provisions per cell did not correlate with changing plant species richness in the environment (Spearman rank correlation: *r* = 0.16, *p* = 0.21) nor with different habitat types (generalized linear model, GLM: *F* = 0.03, *p* = 0.97).

We quantified the sugar content of all 88 samples (to the nearest 0.5 g/g sucrose equivalent) by adding 60 µL of distilled water to each sample, shaking them for 60 min and measuring 2 µL of the supernatant with hand-held refractometers (Eclipse Refractometer, Bellingham + Stanley Ltd., Lawrenceville, NJ, USA) following centrifugation. The sugar concentration (c obtained in %) was converted into *x* (in mg/mL) following Kearns and Inouye [52], using the following polynomial Equation (1):

*x* = −0.0928 + 10.0131 * c + 0.0363 * c^2^ + 0.0002 * c^3^ (1)

this value was then converted into mg sucrose per mg wet larval provision.

Samples were dried overnight at room temperature, before splitting them for two different nutritional analyses and for DNA metabarcoding. We analyzed (i) the amino acid composition of all 88 samples, while (ii) the fatty acid composition was analyzed for only those samples (41) for which we had sufficient amounts (i.e., 20 mg) to perform both analyses (Table S1). As some of our sampled colonies were weak and had only small broods, we only collected few brood cells (and thus not sufficient material for conducting all analyses) because we did not want to severely harm and thus lose our colonies. For 56 samples, we had sufficient amounts to additionally transfer 1–5 mg larval provision to a fresh Eppendorf tube to analyze the number of floral sources within each larval provision using DNA metabarcoding (see below). We required at least 25 mg of substance to conduct all analyses, i.e., amino acid (10 mg), fatty acid (10 mg) and DNA analysis, DNA metabarcoding (1–5 mg), which we only had for 41 of our 88 samples. For samples with fewer amounts, we prioritized the amino acid analysis over the fatty acid analysis, because amino acid composition/protein is typically considered the nutritional component that is most important for bee health/development [53,54]. Because the DNA analysis only required ~1 mg of substance, we could perform DNA metabarcoding for 56 of the 88 samples.

### 2.4. Nutritional Analyses

#### 2.4.1. Amino Acid Analysis

The composition of amino acids of all 88 larval provisions was analyzed by ion exchange chromatography (IEC: Biochrom 20 *plus* amino acid analyzer) following Kriesell et al. [55]. Each sample (12.3 ± 8.4 mg larval provision per sample) was mixed with 200 mL of 6N HCl, boiled for 4 h at 100 °C, cooled down to room temperature and centrifuged (10 min). The supernatant was transferred into a fresh tube. Water was evaporated at 100 °C before the sample was thrice re-dissolved in 200 mL fresh water and centrifuged again. Then, 100 µL were mixed with 12.5% sulphosalicylic acid and extracted for 30 min at ~5 °C, before short mixing and centrifuging (10 min) again. Finally, 100 μL of the supernatant were mixed with 100 μL of sample rarefaction buffer in a fresh microcentrifuge tube, filtered and centrifuged, and analyzed by IEC. We used an external standard (physiological calibration standard, Laborservice Onken GmbH, Gründau, Germany) for amino acid quantification. This standard comprises all amino acids, except for glutamine and asparagine, which quickly deteriorate, and were therefore manually added prior to running standard and samples. Note that our analytical approach destroys tryptophan, which is therefore not considered in our analysis. Interestingly, we did not detect any cysteine in *T. carbonaria* larval provisions, while this amino acid was found to be present in pollen collected and stored by this species, see [44].

#### 2.4.2. Fatty Acid Analysis

To analyze the composition of fatty acids in larval provisions, we adapted the protocol of Rosumek et al. [56] and adjusted it for pollen lipids. All 41 samples (9.6 ± 2.8 mg larval provision per sample) were mixed with 1 mL chloroform/methanol (2:1) and extracted for 24 h on a shaker (Eppendorf, Thermomixer Compact, 40 rpm) at room temperature, before centrifuging and discarding the non-soluble fraction.

Free and bound fatty acids were separated from other compounds on 6ml SiOH polypropylene columns (CHROMABOND^®^, 500mg, Trott, Germany). Columns were conditioned with two column volume equivalents (VE), each of four different solvent mixtures, i.e., chloroform/methanol (2:1), isooctane/ethyl-acetate (10:1), isooctane/ethyl acetate (3:1) and isooctane/ethyl-acetate/acetic acid (75:25:2). Following conditioning, the columns were loaded with the 1 mL sample extract. Di- and triglycerides were eluted with 4 mL isooctane/ethyl acetate (10:1) and 5 mL isooctane/ethyl-acetate (3:1). Free fatty acids were subsequently eluted with 6 mL isooctane/ethyl-acetate/acetic acid (75:25:2). Both fractions were pooled, dried under CO^2^ and resolved in 250 µl dichloromethane/methanol (2:1) before adding 20 µL nonadecanoic acid (in methanol, 0.2 mg/mL) as internal standard and filtering (type 5 μm SVPP, durapore membrane filters, Merck, Darmstadt, Germany). The solvent was completely evaporated (under CO^2^) and the sample re-dissolved in 20 µL derivatizing agent (TMSH, Arcos Organics, NJ, USA). Reagents not previously specified were purchased from Roth (Karlsruhe, Germany) or Sigma-Aldrich (Munich, Germany).

Samples were analyzed with a gas chromatograph coupled to a mass selective spectrometer (GC-MS, Agilent Technologies, 5975 C inert XL MSD). The GC was equipped with a DB-5 fused silica capillary column (30 m × 0.25 mm ID; df = 0.25 μm; Agilent Technologies, USA). The temperature was programmed from an initial of 60 °C for one minute, to 150 °C with a 15 °C/min heating rate, to 260 °C with a 3 °C/min heating rate to 320 °C with a 10 °C/min heating-rate and was then held at 320 °C for 10 min. Helium was used as carrier gas (constant flow of 2.89 mL/min). Injection was carried out at 300 °C in the splitless mode for 1 min. Electron impact mass spectra (EI-MS) were recorded at 70 eV and a source temperature of 250 °C. We used the Windows version of the ChemStation software package (Agilent Technologies, Böblingen, Germany) for data acquisition. Fatty acids were identified based on their mass spectra, retention times and through comparison with synthetic standards (Sigma-Aldrich, Munich, Germany).

### 2.5. Metabarcoding

To assess floral composition and diversity in larval provisions, metabarcoding of larval provisions [57] was performed following the protocol of Sickel et al. [58]: DNA was isolated using the Macherey-Nagel Food Kit (Düren, Germany) and the supplementary protocol of the kit dedicated to pollen samples [59]. The amplification PCR for the ITS2 marker was performed in three separate 10 μL reactions in order to avoid PCR bias. Primers were ITS-S2F [60] and ITS4R [61] but modified for sample multiplexing according to Sickel et al. [58].

Each reaction contained 5 μL 2× Phusion Master Mix (New England Biolabs, Ipswich, MA, USA), 0.33 μM each of the forward and reverse primers, 3.34 μL PCR grade water and 1 μL DNA template. PCR conditions were as follows: initial denaturation at 95 °C for 4 min, 37 cycles of denaturation at 95 °C for 40 s, annealing at 49 °C for 40 s and elongation at 72 °C for 40 s; followed by a final extension step at 72 °C for 5 min. Triplicate reactions of each sample were combined after PCR, and DNA amounts between samples were normalized using the SequalPrep Normalization Plate Kit (Invitrogen GmbH, Darmstadt, Germany). Pooled multiplexed samples were quality controlled using a Bioanalyzer High Sensitivity DNA Chip (Agilent Technologies, Santa Clara, CA, USA) and quantified with the dsDNA High Sensitivity Assay (Life Technologies GmbH, Darmstadt, Germany). Sequencing was performed on the Illumina MiSeq using 2 × 250 cycles v2 chemistry (Illumina Inc., San Diego, CA, USA). Raw sequence data were deposited at the European Nucleotide Archive (ENA, http://www.ebi.ac.uk/ena).

Raw reads were joined, filtered data (<*Q*20, <150 bp, ambiguous base-pairs, chimeras), dereplicated and assigned to biological zero-radius operational taxonomic units (zOTUs) with USEARCH v11 [62]. Sequences were first classified by directs matches (>97% identity) against a global ITS2 reference database [63] created with the bcdatabaser tool [64]. All zOTUs without hits were hierarchically assigned a taxonomy as far as possible with SINTAX against the same reference database. Data were imported into R using the phyloseq package. We filtered Chlorophyta from the dataset and transformed raw read numbers into relative amounts. Genera below a minimum relative abundance of 1% per sample were removed. We calculated floral genus richness per sample based on presence/absence of plant taxa to represent the number of different plant genera from which the pollen originated. We used genus richness to avoid a bias of the abundance of specific taxa as a consequence of the marker-based sequencing approach.

### 2.6. Statistical Analysis

#### 2.6.1. Effects of Surrounding Plant Species Richness and Habitat on Nutrient Contents and Ratios in Larval Nutrition

We composed generalized linear (mixed effect) models to test whether variation in (a) floral genus richness, (b) total nutrient contents (i.e., sucrose, total amino acid (protein) and total fatty acid (fat) content), and (c) nutrient ratios (i.e., the sucrose to protein (S:P), sucrose to fat (S:F), and protein to fat (P:F) ratio) could be explained by the surrounding plant species richness and/or by habitat (i.e., plantation, forest, garden). We used separate analyses for habitat and plant species richness because some plantation sites showed unusually high plant species richness, while some forest sites had very few plant species (more comparable to plantations) [36,44]. Kaluza et al. [44] also showed that plant species richness explained variation, for example, in colony reproduction better than habitat, which is why we analyzed both variables separately. As we had often obtained more than one sample per colony and as some colonies were placed at the same site, we first tested whether this nested structure (sample in colony and site) significantly affected variation in our data by comparing generalized linear mixed effect models (GLMMs, with colony nested in site as random factor) and generalized linear models (GLMs) for each response variable using likelihood ratio tests, as suggested by Zuur et al. [65]. The GLMMs accounting for nestedness did not explain variation significantly better for any of the response variables; thus, we used GLMs throughout. GLMs revealing significant effects of habitat and/or surrounding plant species richness were followed by Tukey’s posthoc tests to assess significant differences between habitats and/or Spearman rank correlation tests to infer the direction of the significant correlation between the response variable and surrounding plant species richness.

#### 2.6.2. Effects of Surrounding Plant Species Richness and Habitat on Nutrient Composition in Larval Nutrition

To assess whether surrounding plant species richness and/or habitat type (i.e., plantation, forest, garden) affected the composition of amino acids and fatty acids, we conducted permutation analyses of variance (PERMANOVA, Adonis command, vegan package, 10,000 permutations) based on Bray-Curtis distance matrices between samples. When significant habitat differences occurred, we used multiple permutation tests (corrected for multiple testing) to assess differences between habitats. For both groups of nutrients, we performed two separate analyses, one based on actual concentrations (in µg/mg pollen) and one based on mass proportions. Mass proportions for each amino and fatty acid were obtained by dividing the concentration of individual amino/fatty acids by the summed concentrations of all amino/fatty acids in a sample. Proportions, therefore, depict ratios of different amino/fatty acids to each other, independent of their concentrations, whereas concentrations display actual amino/fatty acid amounts. Effects of surrounding plant species richness and/or habitat on single amino and fatty acids were then tested using GLMs and Spearman rank correlation tests for both concentrations and proportions (Supplementary Tables S2–S5).

Non-metrical multi-dimensional scaling (NMDS, metaMDS command, vegan package) and environmental fitting (envfit command, vegan package) also based on Bray–Curtis distance matrices between samples were finally used to visualize data in ordination plots.

Prior to testing, all response variables were checked for normality and homoscedasticity and transformed where needed. We then used Gaussian distributions across all GLMs. We used the False Discovery Rate (FDR) to correct for multiple testing of the same data sets. All statistical analyses were performed in R [66].

## 3. Results

### 3.1. Floral Composition and Diversity

**Figure 1:**
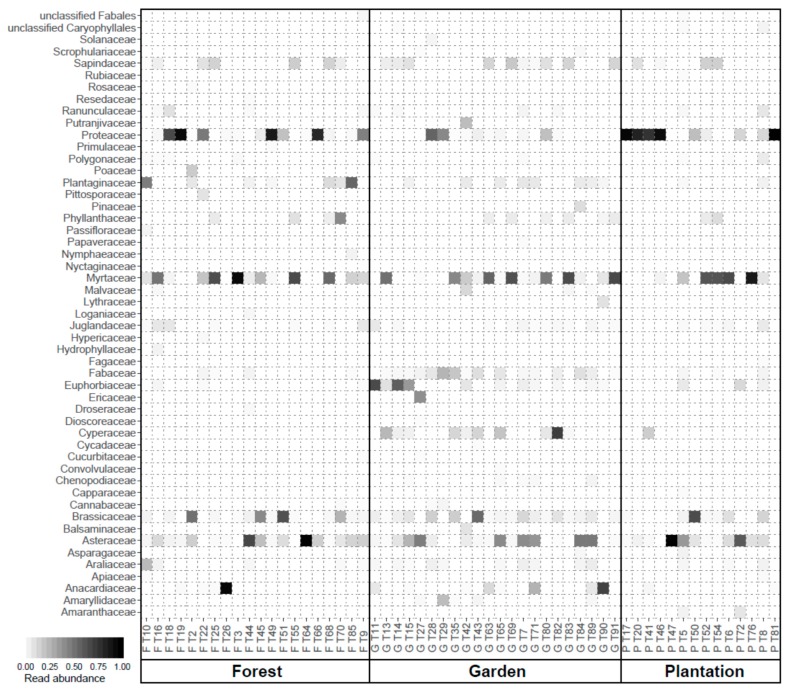
Heatmap of metabarcoding results. The white-to-black gradient of cells represents relative abundances of sequencing read taxonomic assignments on the plant family level (y-axis) for individual sites (x-axis) located in the three different habitats (forest, gardens and plantations), with increasing abundances denoted by darker color. Sequencing reads not assignable to family level are visualized on order level and denoted as “unclassified”.

The number of floral sources in larval provisions of *Tetragonula carbonaria* colonies increased from 9 different plant genera in plantations to 16 in gardens (Table 1, Figure S2). Almost all larval provisions contained plant species of the Proteaceae, Myrtaceae and Asteraceae family, but Proteaceae were more common in forest and plantation than in garden samples (Figure 1 and Figure S3). Larval provisions in gardens often comprised additional plant species from families not consistently found in forest and/or plantation samples, for example, Fabaceae, Euphorbiaceae, Cyperaceae (Figure 1 and Figure S3).

### 3.2. Nutrient Contents and Ratios

Both the sucrose and total fatty acid (i.e., fat) content significantly decreased with increasing surrounding plant species richness, while the protein (i.e., total amino acid content) to fat (P:F) ratio increased on average by more than 40% from plantations to forests and gardens (Table 1). The P:F ratio averaged 7.7:1 in gardens and 7.3:1 in forests, but only 5.7:1 in plantations. Variation among nutrient ratios of different samples was generally high in our study, but lowest for P:F (coefficient of variation (CV): 51%) compared to S:F (CV 54%) and S:P (CV 65%). Variation in protein (i.e., total amino acid) content or other nutrient ratios did not significantly correlate with surrounding plant species richness or habitat (Table 1).

### 3.3. Nutrient Composition

**Figure 2:**
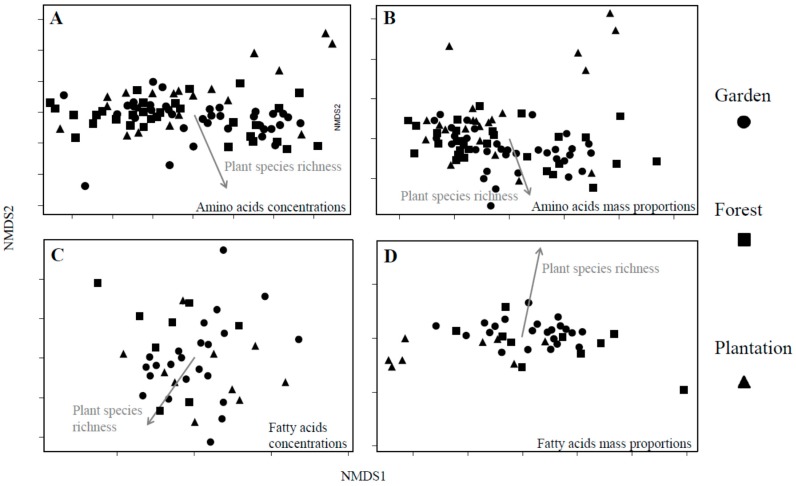
Similarities in the chemical composition of amino acids (**A**,**B**) and fatty acids (**C**,**D**) in larval provisions of *Tetragonula carbonaria* colonies placed in three different habitats (gardens (circles), forests (squares) and plantations (triangles)) and in relation to surrounding plant species richness (grey arrow). Graphs show ordination plots of non-metrical multi-dimensional scaling (NMDS) based on Bray–Curtis distances between samples expressed as concentrations (**A**,**C**) and mass proportions (**B**,**D**) of amino acids and fatty acids. Stress values: A 0.096, B 0.135, C 0.187, D 0.025.

The proportions of explained variance (*R*^2^-values) were generally low for overall compositions of both amino acids and fatty acids in larval provisions, except for relative mass proportions of fatty acids (Table 2). Variation was always explained better by habitat than by plant species richness (Table 2). The overall composition of amino acids as well as most individual amino acids were not affected by surrounding plant species richness or floral genus richness in larval food nor habitat (except for glutamic acid and methionine; Figure 2A, Table 2 and Table S2). In contrast, mass proportions of amino acids were significantly affected by both surrounding plant species richness and habitat (Figure 2B, Table 2). While the proportional amino acid composition was similar for larval provisions produced in gardens and forests, it was different for provisions produced in plantations (Figure 2B and Table S3). Specifically, the proportions of four (out of the 16 analyzed) amino acids (three of them being essential) increased with increasing surrounding plant species richness and were, on average, between 5% and 16% higher in gardens and forests than in plantations (Table S3), while glycine and glutamic acid decreased with increasing surrounding plant species richness on average by 3% (glycine) and 26% (glutamic acid) from plantations to gardens (Table S3).

The overall composition of fatty acids as well as individual fatty acids in larval provisions was affected by both surrounding plant species richness and habitat but not by floral genus richness of provisions (for both concentrations and proportions, Figure 2C,D, Table 2). Again, larval provisions produced by forest and garden colonies were similar to each other but differed from larval provisions produced by colonies located in plantations (Figure 2C,D, Table 2). Concentrations of 2 out of 18 analyzed fatty acids (capric acid and linoleic acid) significantly decreased with increasing plant species richness, for example, on average by 11% (capric acid) and more than 40% (linoleic acid) from gardens to plantations. Concentrations of all other fatty acids did not vary significantly with surrounding plant species richness or habitat type (Table S4). Proportions of 7 out of 18 fatty acids increased with increasing surrounding plant species richness and were, on average, between 30% and 48% higher in gardens or forests than in plantations (Table S5). Only linoleic acid significantly decreased with increasing surrounding plant species richness and was, on average, 40% higher in larval provisions of colonies in plantations than in gardens (Table S5).

## 4. Discussion

Our study indicates that the nutritional quality of larval provisions produced by a highly social Australian bee species changes with the species richness of plants growing in the surrounding habitat. Specifically, we found that, as predicted, in more plant species rich environments, the generalist stingless bee *Tetragonula carbonaria* did not only forage on more plant species [36] but also used floral resources, i.e., pollen and nectar, from more plant species, including additional plant families, to produce larval provisions.

Several nutritional parameters also changed with changing plant species richness in the surrounding habitat. For example, both the sucrose and fat (i.e., total fatty acid) content, as well as the concentration of the omega-6 fatty acid linoleic acid of larval provisions, decreased, while mass proportions of 7 out of 18 fatty acids (including the omega-3 fatty acid linolenic acid) increased. Interestingly, neither the overall protein nor individual amino acid contents in larval provisions showed consistent correlations with habitat- or plant species richness but instead were highly variable across colonies and habitats. This finding suggests that the protein and amino acid content, as well as the amino acid composition of stingless bee larval provisions, may be largely determined by the surrounding plant species community composition. For example, nearly all colonies used floral resources from Proteaceae, Myrtaceae and Asteraceae, albeit in different proportions, to compose larval provisions. Nutritional differences may result from the incorporation of floral resources of additional plant species or the lack of Proteaceae in larval provisions composed at sites with high surrounding plant species richness, such as most garden sites. Some Proteaceae species, for example, of the genus *Banksia*, are known for their comparatively high protein content, while little is known on their fat content [67,68]. Myrtaceae, for example, eucalypts, vary considerably in their protein and fat content both within and between species [67,68]. By selecting specific eucalypt species and or individuals, the bees could, in theory, influence the nutritional quality of allocated floral resources and produced larval provisions. For example, when available, foraging *Tetragonula carbonaria* may select floral resources with specific sucrose and fat content and proportions of specific fatty acids. This would enable the bees to compose larval provisions with a comparatively low fat and sucrose content, a low omega-3:6 ratio and a relatively higher P:F ratio.

This hypothesis relates to findings in bumblebees (i.e., *Bombus impatiens* and *B. terrestris*) and honeybees (*Apis mellifera*). For example, bumblebee foraging rates to specific plants were better explained by pollen P:F than carbohydrate to protein (C:P) ratios [37]. Also, while low concentrations of fat in pollen did not affect pollen consumption by bumblebees, increased concentrations (e.g. above 60 µg/mg pollen) strongly reduced pollen consumption [37,69]. These findings may be explained by the adverse effect of excessive amounts of fat on the bees’ survival. For example, survival of bumblebees was significantly lower when feeding on diets containing low P:F ratios and thus high fat concentrations than the survival of bumblebees feeding on diets containing higher P:F ratios [38,69]. Likewise, survival of honeybees (*Apis mellifera*) decreased with increasing dietary fat content [70]. In particular, increasing concentrations of the omega-6 fatty acid linoleic and oleic acid decreased honeybee survival [70] and impaired their cognitive performance [71], while increasing concentrations of the omega-3 fatty acid linolenic acid supported the bees’ cognitive performance [72]. Moreover, the importance of the omega-6:3 ratio for cognitive performance [71] and the harmful effect of linoleic acid on honeybee survival and cognition may explain why, in our study, the proportion and concentration of linoleic acid decreased and the proportion of linolenic acid increased in larval provisions of stingless bees located in more biodiverse environments. Fatty acids are essential for bee survival and need to be taken up from pollen [72,73,74], but, at the same time, they appear to have severe adverse effects on (honey) bee health and cognition as soon as they exceed a specific concentration in pollen [69,70,71]. This strong dose-dependent effect of fat/fatty acids may explain why honeybee colonies balance deficiencies in fatty acids [39] and may suggest that stingless bees also regulate fat/fatty acid content/ratio when producing larval provisions.

Another previous study found that honeybee larvae survival and development decreased with increasing dietary protein content and tended to be highest on low carbohydrate and low to medium protein diets [75]. This result may explain why the sucrose content of larval provisions in our study also decreased with increasing surrounding plant species richness.

Alternatively, the observed variation in the nutritional composition of larval provisions could also arise from (1) random floral choice among plants in habitats that have different underlying nutrient distributions, or (2) selection of favored plants independent of nutrient ratios that have different abundances in different habitats. Interestingly, the three most abundant plant species in larval provisions (i.e., *Macadamia integrifolia/tetraphylla*, *Raphanus raphanistrum* and *Eucalyptus tereticornis*, see Figure S3) were rare or uncommon at most sites [36] but were found in larval provisions across sites and habitats, rendering random resource selection (1) very unlikely. Given that we do not know the underlying distribution of nutrient availability across all plants in our study habitats, we cannot rule out that the observed changes in the nutritional quality of larval provisions were caused by factors other than nutrient-sensitive selective foraging by bees, such as, for example, floral community composition. A clear limitation of our study is that precise information on the nutritional composition is very limited and largely missing for most of the plant species in our study region. Flowering of different plant species also often occurs irregularly and over variable periods, which renders it very difficult to directly link floral resources found in bee colonies to the surrounding plant community. Moreover, *T. carbonaria* can store floral resources over long periods (of up to several months), which makes it difficult to relate larval provisions to specific time periods. In fact, *T. carbonaria* could, in theory, compensate plant community driven nutrient deficiencies of larval provisions at one time period through adding resources collected at other time periods. Such nutrient balancing over time may represent an advantage of social bees with their long-lived colonies and prolonged activity periods, while solitary species need to maximize the nutritional quality of larval provisions as they forage using resources from the currently available flowering vegetation.

Analyses of pollen nutritional quality (in particular other than crude protein content) are very labor- and cost-intensive and have thus only been conducted for a very limited set of plant species, which shows hardly any overlap with our data set [67,68]. We suggest that efforts should be made to measure variation in chemical profiles (including micro- and macro-nutrients as well as (toxic) plant secondary compounds) of floral resources to be able to better link floral chemistry and availability to pollinator foraging choices and health [76].

In a previous study, we revealed that colony reproduction in *T. carbonaria* also significantly increased with increasing plant species richness and was highest at the most diverse garden and forest sites [44]. The findings from this study may indicate that the beneficial effect of surrounding plant species richness on colony reproductive fitness may be driven by both a continuous resource supply and thus high food quantity over time (as suggested by Kaluza et al. [44]), and also by increased food quality (e.g., through enhancing larval provisions with a low omega-6:3 ratio). Similar field studies on the effect of landscape on colony reproductive output also found a strong effect of resource abundance but no clear effect of resource nutritional quality [9,34]. However, several studies conducted in the laboratory showed a positive correlation between increasing pollen diversity in diets as well as resource nutrient (e.g., pollen protein) content and bee health [11,12,13,14,15]. Our studies further suggest that *T. carbonaria*, like other social bee species, for example, bumblebees [9,34], can compose larval provisions of sufficient quality to sustain proper larval development even in environments with low plant species richness, or that they have a relatively high tolerance for variation in the nutritional quality of larval provisions. The correlations between fat, omega-6 and omega-3 fatty acids, sucrose and (tentatively) the P:F ratio in larval provisions and surrounding plant species richness may nevertheless indicate a positive effect of plant species richness on the quality of composed diets. Limitations in diet nutritional quality might, thus, become more obvious when specific micro- or macro-nutrients are generally limited in the surrounding environment so that they cannot be compensated by diversified consumption [77], food allocation over time, or selective foraging [78]. Our study indicates that such limitations are more likely in less biodiverse environments, such as agricultural monocultures, with potentially adverse effects on wild and managed bees serving as pollinators. Notably, greater floral diversity in larval provisions may alternatively “dilute” pollens of most appropriate nutritional ratios and therefore reduce overall nutritional quality, as key forage plant species may be less abundant or more scattered in habitats with greater floral diversity. Such a dilution effect may even negatively impact bee health, unless they have evolved sophisticated assessment tools to prevent dilution (if possible).

## 5. Conclusions

To conclude, *T. carbonaria* increase overall floral resource diversity intake [36] and the nutritional composition of larval provisions composed by colonies varies with surrounding (floral) plant species richness. While overall amino acid content and amino acid composition show no correlation with habitat or surrounding plant species richness, both the concentration and proportion of fat in general and linoleic acid in particular increased with decreasing plant species richness in the surrounding environment and were highest in macadamia plantations. Given the negative effect of this pollen lipid on both bee health and cognition, this finding may indicate that the bees can face severe health consequences in these low diversity habitats, as was also suggested for honeybees in agricultural areas [72]. Future studies should aim at disentangling the interactive effects of resource diversity, resource quality, resource quantity and resource availability over time to allow a better understanding of how human-induced alterations of floral communities affect the nutritional intake of bees.

## Figures and Tables

**Table 1:** Means ± standard deviation and lower and upper 95% confidence intervals (in brackets) of the number of floral sources (floral genus richness), the sucrose, total fatty acid (fat) and total amino acid (protein) content as well as ratios of sucrose-to-protein (S:P), sucrose-to-fat (S:F) and protein-to-fat (P:F) found in larval provisions (LP) of *Tetragonula carbonaria* colonies placed in three different habitats (gardens (G), forests (F) and plantations (P)). Results shown for generalized linear models (GLM, *F*- and *p*-values) on surrounding plant species richness (richness) and habitat effects, Spearman correlations against surrounding plant species richness (correlation coefficients *r* and *p*-values *p*) and Tukey-posthoc-comparisons for habitat (Tukey). Bold values indicate significant differences and/or correlations and asterisks give significance levels (* *p* < 0.05, ** *p* < 0.01). NS—not significant (*p* > 0.05). NA—not applicable/tested.

	Habitat			GLM (Richness)		Spearman Correlation (Richness)		GLM (Habitat)		Tukey (Habitat, *p*)		
	Garden	Forest	Plantation	*F*	*P*	*r*	*p*	*F*	*p*	G-F	F-P	G-P
Floral genus richness of LP	16.00 ± 6.10 (13.29, 18.71)	11.00 ± 6.76 (7.84, 14.16)	9.43 ± 8.33 (4.62, 14.24)	3.139	0.082	0.258	0.055	**4.635**	0.014 *	NS	NS	0.021 **
Sucrose (mg/mg LP)	0.47 ± 0.20 (0.41, 0.54)	0.57 ± 0.21 (0.50, 0.65)	0.54 ± 0.22 (0.44, 0.64)	**5.565**	0.021 *	**−0.217**	0.042 *	2.016	0.140	NA	NA	NA
Fat (µg/mg LP)	7.22 ± 3.03 (5.91, 8.53)	8.80 ± 6.94 (3.47, 14.13)	9.50 ± 2.00 (7.97, 11.04)	**4.724**	0.036 *	**−0.354**	0.023 *	2.264	0.118	NA	NA	NA
Protein (µg/mg LP)	56.18 ± 24.42 (47.92, 64.44)	63.65 ± 25.81 (54.10, 73.03)	59.53 ± 31.66 (45.12, 73.94)	0.164	0.686	−0.046	0.670	0.815	0.446	NA	NA	NA
Ratio S:P	9.87 ± 6.35 (7.57, 11.55)	8.38 ± 3.28 (8.30, 10.96)	8.25 ± 3.52 (6.81, 16.13)	2.675	0.106	−0.177	0.098	0.386	0.681	NA	NA	NA
Ratio S:F	58.99 ± 33.32 (44.58, 73.39)	46.46 ± 13.08 (36.41, 56.51)	37.54 ± 16.51 (24.85, 50.23)	1.778	0.191	0.150	0.361	1.991	0.151	NA	NA	NA
Ratio P:F	6.73 ± 2.92 (5.47, 7.99)	6.25 ± 2.35 (4.44, 8.05)	4.68 ± 1.39 (3.62, 5.75)	3.590	0.066	0.304	0.053	2.072	0.140	NA	NA	NA

**Table 2:** Results for permutation tests (*R*^2^ and *p*-values) analyzing effects of surrounding plant species richness (Richness) and floral genus richness of larval provisions (Floral richness of LP) and habitat on the composition of amino acids (AA) and fatty acids (FA) (entered as concentrations and mass proportions) in larval provisions (LP) of *Tetragonula carbonaria* colonies placed in three different habitats (gardens (G), forests (F) and plantations (P)). Bold values indicate significant effects and asterisks give significance levels following correction for multiple testing using False Discovery Rate (* *p* < 0.03 (=FDR alpha), ** *p* < 0.01, *** *p* < 0.001). NS—not significant (*p* > 0.05). NA—not applicable/tested.

	Richness		Floral Richness of LP		Habitat		G-F		F-P		G-P	
	*R* ^2^	*p*	*R* ^2^	*p*	*R* ^2^	*p*	*R* ^2^	*p*	*R* ^2^	*p*	*R* ^2^	*p*
AA concentration (µg/mg LP)	0.037	0.147	0.039	0.134	0.022	0.394	NA	NA	NA	NA	NA	NA
AA mass proportion	**0.048**	0.006 **	0.023	0.255	**0.108**	<0.001 ***	NS	NS	**0.092**	0.003 **	**0.134**	<0.001 ***
FA concentration (µg/mg LP)	**0.087**	0.026 *	0.009	0.900	**0.143**	0.018 *	NS	NS	NS	NS	**0.178**	0.005 **
FA mass proportion	0.078	0.063	0.016	0.725	**0.297**	<0.001 ***	NS	NS	**0.342**	0.008 **	**0.326**	<0.001 ***

## Supplementary Materials

The following are available online at https://www.mdpi.com/2075-4450/11/2/125/s1, **Figure S1.** Photographs illustrating (A) hive used to propagate *Tetragonula carbonaria* colonies set up at plant species rich forest site, (B) typical brood spirale of *T. carbonaria* with advancing front as seen when opening hives for propagation and sampling, and (C) top, middle and bottom box of *T. carbonaria* opened in the field to sample brood cells and record colony fitness parameters. **Figure S2.** Plant species number (left) and Shannon diversity (right) of pollen provisions in the three investigated habitats (F = forest, G = gardens, P = plantations). **Figure S3.** Most abundant plant taxa according to metabarcoding read abundance accumulated over all samples in this study. Taxa for which species level assignments were not possible are denoted with “unclassified” and the deepest possible taxonomic level. **Table S1.** Larval provision (LP) samples collected from various colonies at sites situated in three different habitats (forest (F), garden (G) and plantation (P)) differing in the plant species richness (Richness) surrounding colonies and analyzed for their weight, sucrose (S, in mg/ mg LP), protein (P, i.e., total amino acid in µg/mg LP) and fat (F, i.e., total fatty acid in µg/mg LP) content as well as nutrient ratios (S:F, S:P and P:F) and the richness of floral sources in LP (Floral richness). NA indicates missing values. Note that there are some missing values for weights/amounts of larval provisions because we occasionally did not succeed in extracting all material from all collected brood cells and therefore had less material than was originally within brood cells. We only measured amounts of those brood cells were we had managed to extract all material. **Table S2.** Mean concentration (in μg/mg larval provision) ± standard deviation of single amino acids in larval provisions of *Tetragonula carbonaria* colonies placed in three different habitats (gardens (G), forests (F) and plantations (P)). Results shown for generalized linear models (GLM, *F*- and *p*-values) on habitat and surrounding plant species richness (richness) effects, Spearman correlations against surrounding plant species richness (correlation coefficients *r* and *p*-values *p*) and Tukey-posthoc-comparisons (Tukey). Bold values indicate significant differences and/or correlations and asterisks give significance levels (* *p* < 0.05, ** *p* < 0.01, *** *p* < 0.001). NS not significant (*p* > 0.05). NA not applicable/tested. Amino acids essential to honeybees (according to de Groot [53]) are marked in bold and italics. **Table S3.** Mean mass proportion (in% of total amino acid content in larval provision) ± standard deviation of single amino acids in larval provisions of *Tetragonula carbonaria* colonies placed in three different habitats (gardens (G), forests (F) and plantations (P)). Results shown for generalized linear models (GLM, *F*- and *p*-values) on habitat and surrounding plant species richness (richness) effects, Spearman correlations against surrounding plant species richness (correlation coefficients *r* and *p*-values *p*) and Tukey-posthoc-comparisons (Tukey). Bold values indicate significant differences and/or correlations and asterisks give significance levels (* *p* < 0.05, ** *p* < 0.01, *** *p* < 0.001). NS not significant (*p* > 0.05). Amino acids essential to honeybees (according to de Groot [53] are marked in bold and italics. **Table S4.** Mean concentration (in ng/mg larval provision) ± standard deviation of single fatty acids in larval provisions of *Tetragonula carbonaria* colonies placed in three different habitats (gardens (G), forests (F) and plantations (P)). Results shown for generalized linear models (GLM, *F*- and *p*-values) on habitat and surrounding plant species richness (richness) effects, Spearman correlations against surrounding plant species richness (correlation coefficients *r* and *p*-values *p*) and Tukey-posthoc-comparisons (Tukey). Bold values indicate significant differences and/or correlations and asterisks give significance levels (* *p* < 0.05, ** *p* < 0.01, *** *p* < 0.001). NS not significant (*p* > 0.05). Note that linolenic acid and oleic acid could not be entirely separated. **Table S5.** Mean mass proportion (in ^0^/_0000_ of total fatty acid content in larval provision) ± standard deviation of single fatty acids in larval provisions of *Tetragonula carbonaria* colonies placed in three different habitats (gardens (G), forests (F) and plantations (P)). Results shown for generalized linear models (GLM, *F*- and *p*-values) on habitat and surrounding plant species richness (richness) effects, Spearman correlations against surrounding plant species richness (correlation coefficients *r* and *p*-values *p*) and Tukey-posthoc-comparisons (Tukey). Bold values indicate significant differences and/or correlations and asterisks give significance levels (* *p* < 0.05, ** *p* < 0.01, *** *p* < 0.001). NS not significant (*p* > 0.05). Note that linolenic acid and oleic acid could not be entirely separated.
