# Supplementary material for: Floral Species Richness Correlates with Changes in the Nutritional Quality of Larval Diets in a Stingless Bee
Source: Insects. 2020 Feb 15;11(2):125. doi: 10.3390/insects11020125 (PMC7073955; doi:10.3390/insects11020125)
Supplement: Supplementary file 1 [file insects-11-00125-s001.pdf]

**Electronic supplementary material**

**Figure S1.** Photographs illustrating (A) hive used to propagate *Tetragonula carbonaria* colonies set up at plant species rich forest site, (B) typical brood spirale of *T. carbonaria* with advancing front as seen when opening hives for propagation and sampling, and (C) top, middle and bottom box of *T. carbonaria* opened in the field to sample brood cells and record colony fitness parameters.

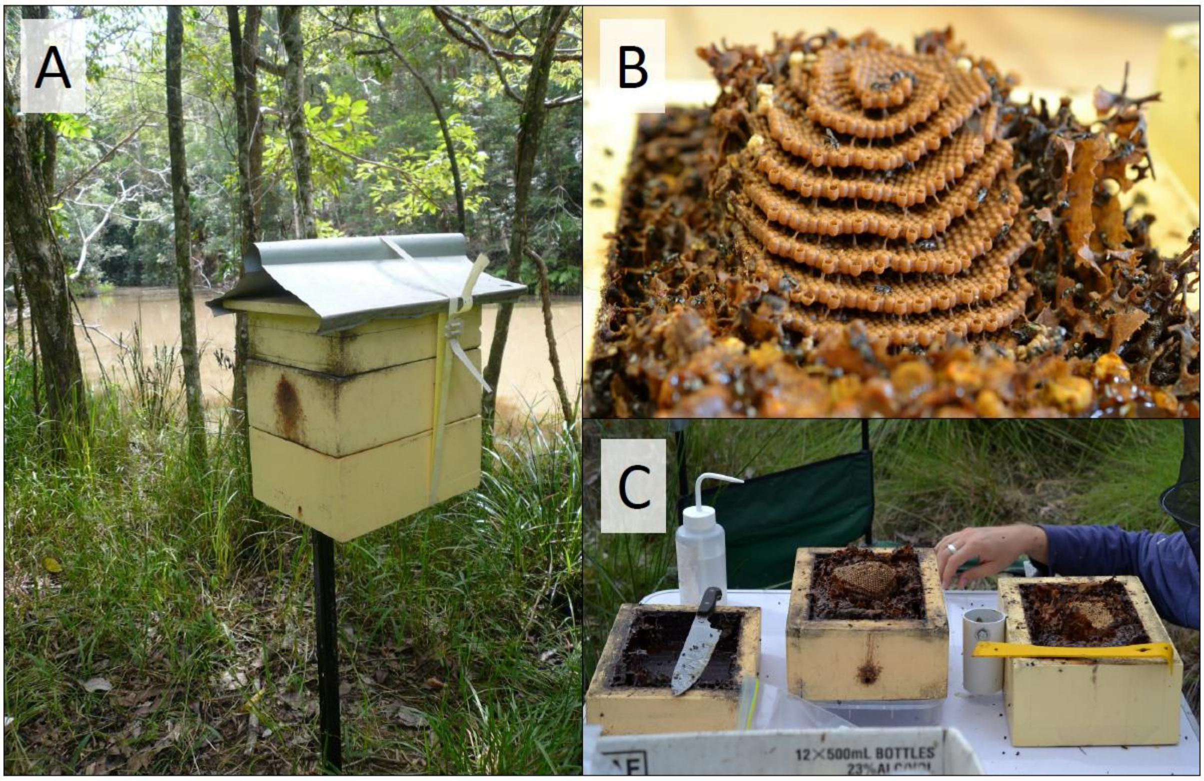

**Figure S2.** Plant species number (left) and Shannon diversity (right) of pollen provisions in the three investigated habitats (F = forest, G = gardens, P = plantations).

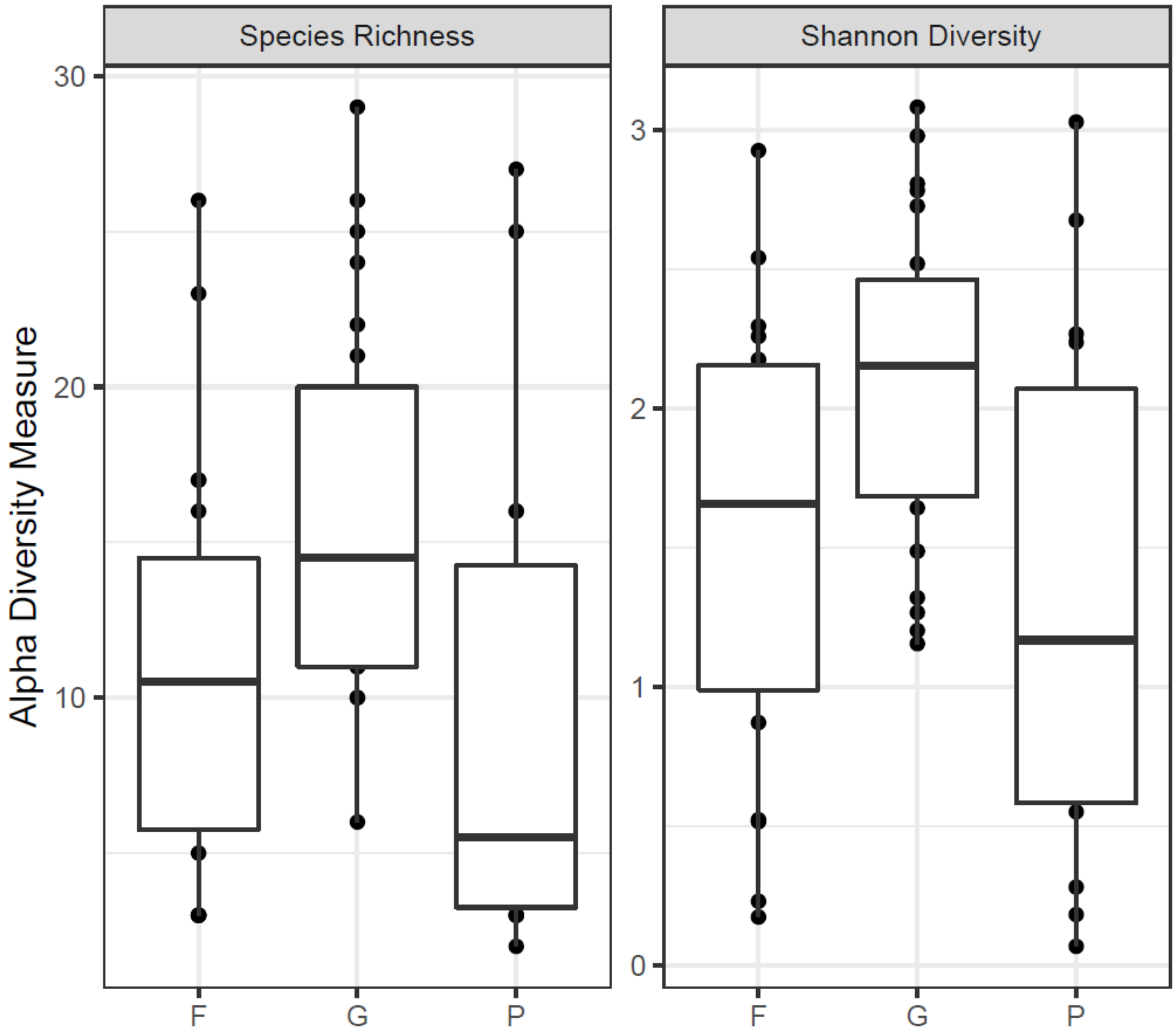

**Figure S3.** Most abundant plant taxa according to metabarcoding read abundance accumulated over all samples in this study. Taxa for which species level assignments were not possible are denoted with "unclassified" and the deepest possible taxonomic level.

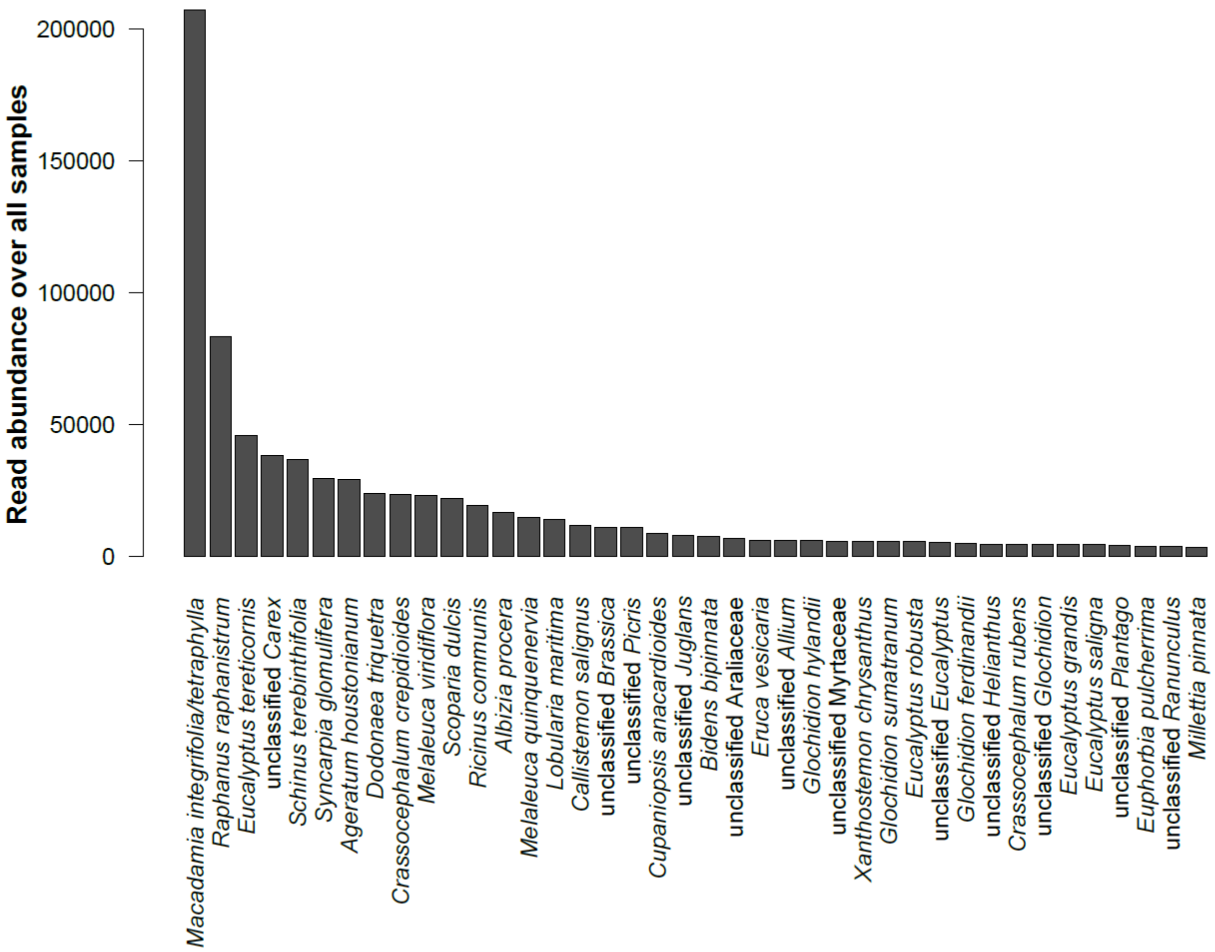

**Table S1.** Larval provision (LP) samples collected from various colonies at sites situated in three different habitats (forest (F), garden (G) and plantation (P)) differing in the plant species richness (Richness) surrounding colonies and analyzed for their weight, sucrose (S, in mg/ mg LP), protein (P, i.e. total amino acid in µg/mg LP) and fat (F, i.e. total fatty acid in µg/mg LP) content as well as nutrient ratios (S:F, S:P and P:F) and the richness of floral sources in LP (Floral richness). NA indicates missing values. Note that there are some missing values for weights/amounts of larval provisions, because we occasionally did not succeed in extracting all material from all collected brood cells and therefore had less material than was originally within brood cells. We only measured amounts of those brood cells were we had managed to extract all material.

| Sample ID | Colony | GPS coordinates | Site | Date of sampling | Rich-ness | Habi-tat | LP weight/ cell | Sucrose [mg/mg] | Protein [µg/mg] | Fat [µg/mg] | Floral richness | S:F | S:P | P:F |
|---|---|---|---|---|---|---|---|---|---|---|---|---|---|---|
| T9 | H101 | 24°38'29"S 152°7'34"E | F1N | 2/21/2012 | 53 | F | NA | 0.57 | 58.49 | 9.12 | 17 | 62.94 | 9.81 | 6.41 |
| T12 | H134 | s.a.a. | F1N | 6/19/2012 | 53 | F | NA | 0.97 | 92.54 | NA | NA | NA | 9.53 | NA |
| T122 | H148 | s.a.a. | F1N | 4/23/2016 | 53 | F | NA | 0.23 | 38.56 | 6.3 | NA | 36.89 | 5.12 | 6.12 |
| T44 | H148 | s.a.a. | F1N | 10/19/2012 | 53 | F | NA | 0.63 | 97.05 | NA | 22 | NA | 5.85 | NA |
| T123 | H209 | s.a.a. | F1N | 4/23/2016 | 53 | F | 5.75 | 0.35 | 40.65 | NA | NA | NA | 7.22 | NA |
| T25 | H209 | s.a.a. | F1N | 8/23/2011 | 53 | F | 7.16 | 0.69 | 55.41 | 26.86 | 15 | 25.71 | 11.32 | 2.06 |
| T45 | H209 | s.a.a. | F1N | 10/19/2012 | 53 | F | NA | 0.81 | 79.85 | NA | 7 | NA | 9.25 | NA |
| T64 | H209 | s.a.a. | F1N | 6/15/2013 | 53 | F | NA | 0.62 | 63.22 | NA | 6 | NA | 8.84 | NA |
| T125 | H184 | 24°46'11"S 152°6'23"E | F2N | 4/23/2016 | 139 | F | 6.29 | 0.26 | 38.15 | 6.23 | NA | 41.76 | 5.78 | 6.12 |
| T49 | H184 | s.a.a. | F2N | 10/17/2012 | 139 | F | NA | 0.74 | 63.26 | NA | 7 | NA | 10.23 | NA |
| T66 | H184 | s.a.a. | F2N | 9/30/2013 | 139 | F | 5.76 | 0.48 | 38.27 | 8.58 | 4 | 56.42 | 11.17 | 4.46 |
| T127 | H194 | s.a.a. | F2N | 4/23/2016 | 139 | F | 7.49 | 0.25 | 26.02 | 4.28 | NA | 57.73 | 7.67 | 6.08 |
| T124 | H215 | s.a.a. | F2N | 4/23/2016 | 139 | F | 8.09 | 0.23 | 60.4 | 5.72 | NA | 40.16 | 3.33 | 10.56 |
| T22 | H215 | s.a.a. | F2N | 8/22/2011 | 139 | F | 6.30 | 0.63 | 66.15 | NA | 11 | NA | 8.56 | NA |
| T10 | H215 | s.a.a. | F2N | 4/1/2012 | 139 | F | NA | 0.62 | 80.79 | NA | 16 | NA | 6.94 | NA |
| T68 | H215 | s.a.a. | F2N | 1/17/2013 | 139 | F | 7.42 | 0.67 | 54.19 | NA | 15 | NA | 10.88 | NA |
| T85 | H103 | 26°43'18"S 153°1'12"E | F3C | 11/14/2013 | 156 | F | 7.08 | 0.49 | 36.53 | NA | 23 | NA | 11.47 | NA |
| T3 | H191 | s.a.a. | F3C | 1/31/2012 | 156 | F | NA | 0.68 | 82.72 | NA | 6 | NA | 7.2 | NA |
| T26 | H207 | s.a.a. | F3C | 9/6/2011 | 156 | F | NA | 0.65 | 53.35 | NA | 9 | NA | 11.16 | NA |
| T16 | H207 | s.a.a. | F3C | 7/5/2012 | 156 | F | NA | 0.72 | 91.94 | NA | 21 | NA | 7.12 | NA |
| T4 | H272 | s.a.a. | F3C | 1/31/2012 | 156 | F | NA | 0.75 | 115.57 | NA | NA | NA | 5.98 | NA |
| T55 | H272 | s.a.a. | F3C | 3/14/2013 | 156 | F | NA | 0.8 | 104.36 | NA | 12 | NA | 6.89 | NA |
| T19 | H113 | 26°50'2"S 152°54'57"E | F4C | 7/24/2012 | 173 | F | 7.12 | 0.61 | 48 | NA | 4 | NA | 11.35 | NA |
| T101 | H150 | s.a.a. | F4C | 4/8/2016 | 173 | F | 7.33 | 0.24 | 63.73 | NA | NA | NA | 3.38 | NA |
| T2 | H150 | s.a.a. | F4C | 1/20/2012 | 173 | F | NA | 0.65 | 32.85 | NA | 8 | NA | 17.18 | NA |
| T115 | H169 | s.a.a. | F4C | 4/8/2016 | 173 | F | NA | 0.32 | 32.05 | 5.35 | NA | 60.11 | 8.37 | 5.99 |
| T117 | H177 | s.a.a. | F4C | 4/8/2016 | 173 | F | 5.60 | 0.25 | 57.11 | 6.78 | NA | 36.45 | 3.85 | 8.42 |
| T51 | H177 | s.a.a. | F4C | 11/7/2012 | 173 | F | 6.86 | 0.69 | 125.76 | NA | 4 | NA | 4.98 | NA |
| T67 | H177 | s.a.a. | F4C | 11/15/2013 | 173 | F | NA | 0.79 | 81.1 | NA | NA | NA | 8.72 | NA |
| T18 | H274 | s.a.a. | F4C | 7/20/2012 | 173 | F | 9.20 | 0.6 | 51.01 | NA | 10 | NA | 10.26 | NA |
| T70 | H274 | s.a.a. | F4C | 3/5/2013 | 173 | F | 6.30 | 0.71 | 41.4 | NA | 9 | NA | 14.95 | NA |
| T29 | H118 | 24°50'26"S 152°28'26"E | G1N | 9/17/2011 | 259 | G | 6.22 | 0.53 | 66.01 | 7.28 | 7 | 72.66 | 7.33 | 9.07 |
| T7 | H199 | s.a.a. | G1N | 2/21/2012 | 288 | G | 5.57 | 0.64 | 20.96 | 3.67 | 20 | 175.38 | 25.42 | 5.71 |
| T71 | H199 | s.a.a. | G1N | 3/7/2013 | 288 | G | 7.20 | 0.67 | 96.3 | NA | 19 | NA | 6.28 | NA |
| T121 | H231 | s.a.a. | G1N | 4/28/2016 | 288 | G | 7.91 | 0.22 | 30.65 | 6.24 | NA | 34.96 | 5.89 | 4.91 |

| | | | | | | | | | | | | | | |
|---|---|---|---|---|---|---|---|---|---|---|---|---|---|---|
| T28 | H243 | s.a.a. | G1N | 9/17/2011 | 288 | G | 6.38 | 0.64 | 108.64 | NA | 12 | NA | 5.35 | NA |
| T63 | H279 | s.a.a. | G1N | 6/14/2013 | 288 | G | 6.68 | 0.47 | 51.01 | 9.3 | 14 | 50.25 | 8.01 | 5.48 |
| T132 | H347 | s.a.a. | G1N | 4/28/2016 | 288 | G | 7.11 | 0.21 | 40.47 | 6.43 | NA | 33.44 | 4.39 | 6.29 |
| T43 | H108 | 26°40'42"S 153° 6'40"E | G2C | 11/5/2012 | 239 | G | 4.63 | 0.69 | 78.62 | NA | 11 | NA | 7.93 | NA |
| T65 | H129 | s.a.a. | G2C | 6/27/2013 | 278 | G | 7.85 | 0.76 | 62.22 | NA | 19 | NA | 10.84 | NA |
| T91 | H137 | s.a.a. | G2C | 3/9/2016 | 239 | G | 7.16 | 0.48 | 29.48 | 6.71 | 14 | 71.94 | 13.97 | 4.39 |
| T27 | H138 | s.a.a. | G2C | 9/6/2011 | 239 | G | 6.22 | 0.65 | 96.75 | NA | 12 | NA | 5.95 | NA |
| T84 | H138 | s.a.a. | G2C | 11/14/2013 | 239 | G | 6.51 | 0.47 | 44.59 | NA | 18 | NA | 9.46 | NA |
| T35 | H170 | s.a.a. | G2C | 9/28/2011 | 278 | G | 5.98 | 0.56 | 64.93 | 4.91 | 9 | 114.93 | 7.79 | 13.22 |
| T89 | H172 | s.a.a. | G2C | 3/9/2016 | 239 | G | 7.62 | 0.61 | 39.98 | 7.14 | 18 | 85.8 | 13.54 | 5.6 |
| T90 | H370 | s.a.a. | G2C | 3/9/2016 | 278 | G | 6.85 | 0.75 | 32.2 | 18.61 | 13 | 40.42 | 20.52 | 1.73 |
| T113 | H371 | s.a.a. | G2C | 5/2/2016 | 239 | G | 4.46 | 0.31 | 35.72 | 6.96 | NA | 44.28 | 7.21 | 5.13 |
| T98 | H393 | s.a.a. | G2C | 5/2/2016 | 278 | G | 7.37 | 0.23 | 48.7 | 5.97 | NA | 38.5 | 4.15 | 8.16 |
| T103 | H396 | s.a.a. | G2C | 5/2/2016 | 239 | G | 7.60 | 0.22 | 47.98 | 4.8 | NA | 45.98 | 4.05 | 10 |
| T24 | H107 | 27°29'0"S 153° 0'16"E | G3S | 8/10/2011 | 340 | G | NA | 0.81 | 61.47 | NA | NA | NA | 11.82 | NA |
| T83 | H107 | s.a.a. | G3S | 11/6/2013 | 340 | G | 7.45 | 0.47 | 49.8 | 10.78 | 14 | 43.94 | 8.51 | 4.62 |
| T13 | H152 | s.a.a. | G3S | 6/26/2012 | 411 | G | 9.18 | 0.37 | 54.84 | 3.68 | 14 | 99.4 | 5.97 | 14.9 |
| T42 | H233 | s.a.a. | G3S | 12/12/2012 | 411 | G | 6.12 | 0.64 | 104.67 | NA | 15 | NA | 5.52 | NA |
| T80 | H233 | s.a.a. | G3S | 7/9/2013 | 411 | G | 6.62 | 0.43 | 50.93 | 9 | 14 | 48.05 | 7.37 | 5.66 |
| T105 | H320 | s.a.a. | G3S | 5/6/2016 | 411 | G | 7.12 | 0.25 | 31.14 | 5.08 | NA | 48.73 | 6.56 | 6.13 |
| T95 | H422 | s.a.a. | G3S | 5/6/2016 | 340 | G | NA | 0.17 | 66.05 | NA | NA | NA | 2.18 | NA |
| T106 | H123 | 27°29'46"S 153° 1'24"E | G4S | 4/15/2016 | 411 | G | 5.73 | 0.28 | 50.76 | 7.02 | NA | 39.53 | 4.75 | 7.23 |
| T14 | H162 | s.a.a. | G4S | 6/26/2012 | 398 | G | 7.91 | 0.49 | 36.7 | 8.21 | 14 | 59.44 | 11.55 | 4.47 |
| T107 | H211 | s.a.a. | G4S | 4/15/2016 | 411 | G | 7.46 | 0.25 | 44.46 | 6.96 | NA | 35.73 | 4.84 | 6.39 |
| T23 | H211 | s.a.a. | G4S | 8/10/2011 | 411 | G | 6.24 | 0.7 | 83.38 | NA | NA | NA | 7.63 | NA |
| T82 | H211 | s.a.a. | G4S | 10/19/2013 | 411 | G | 6.93 | 0.48 | 52.67 | 8.58 | 9 | 55.47 | 7.9 | 6.14 |
| T11 | H226 | s.a.a. | G4S | 4/3/2012 | 398 | G | 11.42 | 0.46 | 81.9 | NA | 9 | NA | 5.01 | NA |
| T15 | H266 | s.a.a. | G4S | 6/26/2012 | 398 | G | 7.42 | 0.65 | 28.14 | NA | 17 | NA | 20.22 | NA |
| T69 | H266 | s.a.a. | G4S | 1/24/2013 | 398 | G | 6.24 | 0.75 | 109.29 | NA | 14 | NA | 6.16 | NA |
| T114 | H299 | s.a.a. | G4S | 5/6/2016 | 398 | G | 7.79 | 0.23 | 28.17 | 5.46 | NA | 41.87 | 6.7 | 5.16 |
| T112 | H363 | s.a.a. | G4S | 4/15/2016 | 411 | G | 7.07 | 0.24 | 51.33 | 5.8 | NA | 41.39 | 4.08 | 8.85 |
| T111 | H404 | s.a.a. | G4S | 5/6/2016 | 398 | G | 6.30 | 0.26 | 41.56 | 7.52 | NA | 34.56 | 5.45 | 5.53 |
| T21 | H164 | 24°46'25"S 152°15'10"E | P1N | 8/1/2012 | 40 | P | NA | 0.88 | 17.05 | NA | NA | NA | 45.84 | NA |
| T72 | H164 | s.a.a. | P1N | 3/8/2013 | 40 | P | NA | 0.76 | 34.53 | NA | 6 | NA | 18.49 | NA |
| T5 | H237 | s.a.a. | P1N | 2/20/2012 | 40 | P | 6.32 | 0.66 | 46.09 | 12.92 | 18 | 50.83 | 11.99 | 3.57 |
| T6 | H153 | 24°47'5"S 152°15'25"E | P2N | 2/20/2012 | 51 | P | 4.54 | 0.66 | 77.1 | NA | 10 | NA | 7.62 | NA |
| T126 | H196 | s.a.a. | P2N | 4/23/2016 | 51 | P | 6.57 | 0.24 | 45.07 | 9.38 | NA | 26.02 | 4.31 | 4.81 |
| T52 | H225 | s.a.a. | P2N | 11/24/2012 | 51 | P | NA | 0.91 | 61.53 | NA | 14 | NA | 12.99 | NA |
| T131 | H242 | s.a.a. | P2N | 4/23/2016 | 51 | P | NA | 0.35 | 52.75 | 7.21 | NA | 48.41 | 5.29 | 7.32 |
| T76 | H242 | s.a.a. | P2N | 3/30/2013 | 51 | P | NA | 0.58 | 65.41 | NA | 6 | NA | 7.84 | NA |
| T81 | H135 | 24°47'9"S 152°16'6"E | P3N | 10/10/2013 | 134 | P | NA | 0.61 | 37.12 | NA | 2 | NA | 14.22 | NA |
| T128 | H160 | s.a.a. | P3N | 4/24/2016 | 134 | P | 8.02 | 0.22 | 39.43 | 10.02 | NA | 21.7 | 4.46 | 3.94 |
| T41 | H160 | s.a.a. | P3N | 9/21/2011 | 134 | P | 6.10 | 0.68 | 112.71 | NA | 5 | NA | 5.45 | NA |
| T8 | H160 | s.a.a. | P3N | 2/21/2012 | 134 | P | 6.38 | 0.51 | 44.69 | NA | 21 | NA | 9.58 | NA |
| T47 | H160 | s.a.a. | P3N | 10/16/2012 | 134 | P | 6.32 | 0.75 | 142.04 | NA | 5 | NA | 4.77 | NA |
| T46 | H165 | s.a.a. | P3N | 10/16/2012 | 134 | P | NA | 0.64 | 113.59 | NA | 3 | NA | 5.18 | NA |

| | | | | | | | | | | | | | | |
|---|---|---|---|---|---|---|---|---|---|---|---|---|---|---|
| T129 | H225 | s.a.a. | P3N | 4/24/2016 | 134 | P | 6.65 | 0.25 | 33.31 | 9.06 | NA | 27.36 | 5.73 | 3.68 |
| T20 | H244 | s.a.a. | P3N | 8/1/2012 | 134 | P | 8.48 | 0.63 | 90.04 | NA | 5 | NA | 6.28 | NA |
| T130 | H308 | s.a.a. | P3N | 4/24/2016 | 134 | P | 7.54 | 0.23 | 48.88 | 9.7 | NA | 24.17 | 4.04 | 5.04 |
| T17 | H168 | 26°53'13"S 152°56'5"E | P4C | 7/16/2012 | 69 | P | 7.11 | 0.49 | 45.88 | 7.06 | 5 | 69.22 | 9.18 | 6.5 |
| T108 | H174 | s.a.a. | P4C | 5/5/2016 | 69 | P | 7.89 | 0.2 | 31.16 | 8.11 | NA | 25 | 5.5 | 3.84 |
| T54 | H178 | s.a.a. | P4C | 3/5/2013 | 69 | P | 7.90 | 0.55 | 41.71 | 12.07 | 14 | 45.18 | 11.47 | 3.46 |
| T50 | H251 | s.a.a. | P4C | 10/23/2012 | 69 | P | 6.22 | 0.62 | 70.06 | NA | 4 | NA | 7.89 | NA |

**Table S2.** Mean concentration (in µg/mg larval provision) ± standard deviation of single amino acids in larval provisions of *Tetragonula carbonaria* colonies placed in three different habitats (gardens (G), forests (F) and plantations (P)). Results shown for generalized linear models (GLM, *F*- and *p*-values) on habitat and surrounding plant species richness (richness) effects, Spearman correlations against surrounding plant species richness (correlation coefficients *r* and *p*-values *p*) and Tukey-posthoc-comparisons (Tukey). Bold values indicate significant differences and/or correlations and asterisks give significance levels (**p* < 0.05, ***p* < 0.01, ****p* < 0.001). NS not significant (*p* > 0.05). NA not applicable/tested. Amino acids essential to honeybees (according to de Groot (1953)) are marked in bold and italics.

| | Habitat | | | GLM (richness) | | Spearman correlation (richness) | | GLM (habitat) | | Tukey (habitat, *p*) | | |
|---|---|---|---|---|---|---|---|---|---|---|---|---|
| | Garden | Forest | Plantation | *F* | *p* | *r* | *p* | *F* | *p* | G-F | F-P | G-P |
| Asparagine | 8.78±3.97 | 9.50±3.92 | 8.89±4.86 | 0.004 | 0.950 | 0.010 | 0.923 | 0.266 | 0.767 | NA | NA | NA |
| ***Threonine*** | 2.95±1.62 | 3.55±1.76 | 3.33±2.11 | 0.488 | 0.487 | -0.072 | 0.505 | 0.957 | 0.388 | NA | NA | NA |
| Serine | 4.91±2.01 | 5.43±2.03 | 5.18±2.53 | 0.049 | 0.825 | -0.029 | 0.790 | 0.495 | 0.612 | NA | NA | NA |
| Glutamic acid | 3.57±1.88 | 4.31±2.17 | 4.93±2.63 | 2.180 | 0.144 | -0.222 | **0.038*** | 2.736 | 0.071 | NA | NA | NA |
| Proline | 4.69±1.63 | 5.28±1.66 | 5.06±2.23 | 0.183 | 0.670 | -0.055 | 0.612 | 0.924 | 0.401 | NA | NA | NA |
| Glycine | 3.19±1.30 | 3.67±1.51 | 3.49±1.74 | 0.376 | 0.541 | -0.087 | 0.420 | 0.874 | 0.421 | NA | NA | NA |
| Alanine | 3.40±1.55 | 4.04±1.66 | 3.53±1.98 | 0.294 | 0.589 | -0.057 | 0.598 | 1.246 | 0.239 | NA | NA | NA |
| ***Valine*** | 1.95±1.06 | 2.24±1.07 | 2.14±1.29 | 0.139 | 0.710 | -0.056 | 0.603 | 0.558 | 0.574 | NA | NA | NA |
| ***Methionine*** | 0.76±0.34 | 0.61±0.34 | 0.61±0.31 | 7.258 | **0.008**** | 0.281 | **0.008**** | 2.254 | 0.111 | NA | NA | NA |
| ***Isoleucine*** | 1.71±0.77 | 1.90±0.78 | 1.77±0.10 | 0.017 | 0.896 | -0.005 | 0.966 | 0.480 | 0.620 | NA | NA | NA |
| ***Leucine*** | 5.58±2.60 | 6.28±2.61 | 5.84±3.25 | 0.107 | 0.744 | -0.036 | 0.740 | 0.543 | 0.583 | NA | NA | NA |
| Tyrosine | 1.78±0.92 | 2.15±1.00 | 1.95±1.17 | 0.445 | 0.507 | -0.071 | 0.513 | 1.132 | 0.327 | NA | NA | NA |
| ***Phenylalanine*** | 2.23±1.06 | 2.56±1.14 | 2.27±1.39 | 0.093 | 0.762 | -0.020 | 0.850 | 0.733 | 0.484 | NA | NA | NA |
| ***Lysine*** | 5.32±2.07 | 6.03±2.56 | 5.38±3.04 | 0.005 | 0.944 | 0.031 | 0.777 | 0.763 | 0.470 | NA | NA | NA |
| ***Histidine*** | 1.63±0.63 | 1.87±0.71 | 1.66±0.88 | 0.033 | 0.857 | 0.016 | 0.880 | 1.072 | 0.347 | NA | NA | NA |
| ***Arginine*** | 3.75±1.56 | 4.14±1.70 | 3.48±2.04 | 0.150 | 0.700 | 0.067 | 0.535 | 0.969 | 0.384 | NA | NA | NA |

**Table S3.** Mean mass proportion (in % of total amino acid content in larval provision) ± standard deviation of single amino acids in larval provisions of *Tetragonula carbonaria* colonies placed in three different habitats (gardens (G), forests (F) and plantations (P)). Results shown for generalized linear models (GLM, *F*- and *p*-values) on habitat and surrounding plant species richness (richness) effects, Spearman correlations against surrounding plant species richness (correlation coefficients *r* and *p*-values *p*) and Tukey-posthoc-comparisons (Tukey). Bold values indicate significant differences and/or correlations and asterisks give significance levels (**p* < 0.05, ***p* < 0.01, ****p* < 0.001). NS not significant (*p* > 0.05). Amino acids essential to honeybees (according to de Groot (1953) are marked in bold and italics.

| | Habitat | | | GLM (richness) | | Spearman correlation (richness) | | GLM (habitat) | | Tukey (habitat, *p*) | | |
|---|---|---|---|---|---|---|---|---|---|---|---|---|
| | Garden | Forest | Plantation | *F* | *p* | *r* | *p* | *F* | *p* | G-F | F-P | G-P |
| Asparagine | 15.56±0.86 | 14.87±0.81 | 14.78±0.84 | 10.467 | **0.002**** | 0.305 | **0.004**** | 7.189 | **0.001**** | NS | NS | **0.003**** |
| ***Threonine*** | 5.06±0.80 | 5.39±0.80 | 5.39±0.64 | 3.436 | 0.067 | -0.141 | 0.192 | 1.936 | 0.151 | NA | NA | NA |
| Serine | 8.83±0.44 | 8.66±0.48 | 8.84±0.48 | 1.083 | 0.301 | 0.113 | 0.295 | 1.401 | 0.252 | NA | NA | NA |
| Glutamic acid | 6.17±0.96 | 6.55±0.96 | 8.46±2.10 | 13.015 | **<0.001***** | -0.411 | **<0.001***** | 21.232 | **<0.001***** | NS | **<0.001***** | **<0.001***** |
| Proline | 8.64±1.12 | 8.74±1.86 | 8.87±1.72 | 0.078 | 0.781 | 0.063 | 0.561 | 0.149 | 0.862 | NA | NA | NA |
| Glycine | 5.76±0.38 | 5.78±0.31 | 5.93±0.37 | 3.047 | 0.084 | -0.219 | **0.040*** | 1.636 | 0.201 | NA | NA | NA |
| Alanine | 6.03±0.37 | 6.36±0.43 | 5.89±0.43 | 2.487 | 0.119 | -0.134 | 0.211 | 9.600 | **<0.001***** | **0.004**** | **<0.001***** | NS |
| ***Valine*** | 3.35±0.55 | 3.43±0.44 | 3.49±0.42 | 0.667 | 0.417 | -0.024 | 0.824 | 0.588 | 0.558 | NA | NA | NA |
| ***Methionine*** | 1.43±0.60 | 1.09±0.67 | 1.16±0.67 | 6.383 | **0.013*** | 0.267 | **0.012*** | 2.602 | 0.080 | NA | NA | NA |
| ***Isoleucine*** | 3.03±0.20 | 2.99±0.24 | 2.94±0.21 | 1.762 | 0.188 | 0.158 | 0.141 | 1.040 | 0.358 | NA | NA | NA |
| ***Leucine*** | 9.85±0.67 | 9.84±1.00 | 9.73±0.90 | 0.021 | 0.885 | 0.007 | 0.948 | 0.150 | 0.861 | NA | NA | NA |
| Tyrosine | 3.08±0.42 | 3.31±0.34 | 3.18±0.38 | 3.741 | 0.056 | -0.147 | 0.171 | 2.838 | 0.064 | NA | NA | NA |
| ***Phenylalanine*** | 3.91±0.27 | 3.98±0.28 | 3.73±0.36 | 0.000 | 0.977 | 0.007 | 0.952 | 4.456 | **0.014*** | NS | **0.012*** | NS |
| ***Lysine*** | 9.64±0.86 | 9.49±1.01 | 8.97±1.37 | 3.110 | 0.081 | 0.198 | 0.065 | 2.730 | 0.071 | NA | NA | NA |
| ***Histidine*** | 2.94±0.31 | 3.00±0.41 | 2.79±0.30 | 0.805 | 0.372 | 0.078 | 0.471 | 2.294 | 0.107 | NA | NA | NA |
| ***Arginine*** | 6.70±0.41 | 6.52±0.46 | 5.75±0.64 | 20.360 | **<0.001***** | 0.441 | **<0.001***** | 26.112 | **<0.001***** | NS | **<0.001***** | **<0.001***** |

**Table S4.** Mean concentration (in ng/mg larval provision) ± standard deviation of single fatty acids in larval provisions of *Tetragonula carbonaria* colonies placed in three different habitats (gardens (G), forests (F) and plantations (P)). Results shown for generalized linear models (GLM, *F*- and *p*-values) on habitat and surrounding plant species richness (richness) effects, Spearman correlations against surrounding plant species richness (correlation coefficients *r* and *p*-values *p*) and Tukey-posthoc-comparisons (Tukey). Bold values indicate significant differences and/or correlations and asterisks give significance levels (*$p$ < 0.05, **$p$ < 0.01, ***$p$ < 0.001). NS not significant ($p$ > 0.05). Note that linolenic acid and oleic acid could not be entirely separated.

| | Habitat | | | GLM (richness) | | Spearman correlation (richness) | | GLM (habitat) | | Tukey (habitat, *p*) | | |
|---|---|---|---|---|---|---|---|---|---|---|---|---|
| | Garden | Forest | Plantation | *F* | *p* | *r* | *p* | *F* | *p* | G-F | F-P | G-P |
| Caprylic acid | 1.51±1.24 | 1.76±1.73 | 1.36±0.58 | 0.727 | 0.399 | -0.114 | 0.479 | 0.230 | 0.796 | NA | NA | NA |
| Pelargonic acid | 1.35±0.68 | 1.93±1.99 | 1.46±0.64 | 3.201 | 0.084 | -0.227 | 0.154 | 0.912 | 0.410 | NA | NA | NA |
| Adipic acid | 0.49±0.48 | 0.81±0.73 | 0.78±0.92 | 5.579 | **0.023*** | -0.247 | 0.119 | 1.083 | 0.349 | NA | NA | NA |
| Capric acid | 23.08±13.25 | 31.18±21.84 | 26.04±9.75 | 4.022 | 0.052 | -0.395 | **0.011*** | 0.964 | 0.391 | NA | NA | NA |
| Undecanoic acid | 0.45±0.23 | 0.75±0.95 | 0.53±0.22 | 3.127 | 0.085 | -0.248 | 0.118 | 1.181 | 0.318 | NA | NA | NA |
| Lauric acid | 25.42±11.96 | 31.24±17.62 | 25.56±9.39 | 1.715 | 0.198 | -0.185 | 0.248 | 0.703 | 0.501 | NA | NA | NA |
| Myristic acid | 125.09±50.28 | 158.57±69.72 | 133.42±38.50 | 2.213 | 0.145 | -0.179 | 0.262 | 1.296 | 0.285 | NA | NA | NA |
| Pentadecanoic acid | 2.17±1.04 | 2.82±1.82 | 2.54±1.07 | 3.225 | 0.080 | -0.241 | 0.130 | 0.952 | 0.395 | NA | NA | NA |
| Palmitic acid | 1837.38±807.89 | 2220.21±1238.29 | 1825.42±430.97 | 1.251 | 0.270 | -0.130 | 0.417 | 0.709 | 0.499 | NA | NA | NA |
| cis-10-Heptadecenoic acid | 2.24±0.97 | 2.78±0.73 | 2.85±0.98 | 3.038 | 0.089 | -0.201 | 0.208 | 1.959 | 0.155 | NA | NA | NA |
| Stearic acid | 1666.59±694.37 | 2041.58±942.56 | 1725.73±468.82 | 1.553 | 0.220 | -0.160 | 0.317 | 0.902 | 0.414 | NA | NA | NA |
| Nonadecanoic acid | 6.61±4.02 | 7.60±5.77 | 5.65±1.92 | 0.046 | 0.831 | -0.052 | 0.746 | 0.499 | 0.611 | NA | NA | NA |
| Arachidic acid | 105.06±46.50 | 127.76±47.49 | 119.80±35.00 | 2.194 | 0.147 | -0.217 | 0.173 | 0.964 | 0.391 | NA | NA | NA |
| Behenic acid | 33.80±16.91 | 38.43±25.52 | 36.85±12.97 | 0.527 | 0.472 | -0.095 | 0.555 | 0.267 | 0.790 | NA | NA | NA |
| Lignoceric acid | 1.56±1.03 | 1.59±1.04 | 1.82±0.81 | 1.246 | 0.271 | -0.244 | 0.124 | 0.238 | 0.789 | NA | NA | NA |
| Oleic acid | 2.29±1.03 | 2.80±2.10 | 2.74±0.89 | 2.315 | 0.136 | -0.227 | 0.154 | 0.682 | 0.512 | NA | NA | NA |
| Linoleic acid | 2567.93±1230.42 | 3223.98±3815.51 | 4762.69±1735.89 | 6.721 | **0.013*** | -0.384 | **0.013*** | 3.406 | **0.044*** | NS | NS | **0.034*** |
| Linolenic acid | 818.60±362.59 | 905.01±867.32 | 828.71±251.48 | 0.441 | 0.511 | 0.003 | 0.987 | 0.100 | 0.905 | NA | NA | NA |

**Table S5.** Mean mass proportion (in $^{0}/_{0000}$ of total fatty acid content in larval provision) ± standard deviation of single fatty acids in larval provisions of *Tetragonula carbonaria* colonies placed in three different habitats (gardens (G), forests (F) and plantations (P)). Results shown for generalized linear models (GLM, *F*- and *p*-values) on habitat and surrounding plant species richness (richness) effects, Spearman correlations against surrounding plant species richness (correlation coefficients *r* and *p*-values *p*) and Tukey-posthoc-comparisons (Tukey). Bold values indicate significant differences and/or correlations and asterisks give significance levels (**p* < 0.05, ***p* < 0.01, ****p* < 0.001). NS not significant (*p* > 0.05). Note that linolenic acid and oleic acid could not be entirely separated.

| | Habitat | | | GLM (richness) | | Spearman correlation (richness) | | GLM (habitat) | | Tukey (habitat, *p*) | | |
|---|---|---|---|---|---|---|---|---|---|---|---|---|
| | Garden | Forest | Plantation | *F* | *p* | *r* | *p* | *F* | *p* | G-F | F-P | G-P |
| Caprylic acid | 1.98±0.90 | 1.96±1.17 | 1.41±0.48 | 0.144 | 0.706 | 0.098 | 0.540 | 1.369 | 0.267 | NA | NA | NA |
| Pelargonic acid | 1.95±0.86 | 1.99±0.83 | 1.52±0.44 | <0.001 | 0.988 | -0.041 | 0.800 | 1.153 | 0.326 | NA | NA | NA |
| Adipic acid | 0.70±0.63 | 0.92±0.67 | 0.75±0.77 | 2.033 | 0.162 | -0.151 | 0.345 | 0.345 | 0.710 | NA | NA | NA |
| Capric acid | 31.86±9.89 | 37.13±9.56 | 27.98±11.11 | 0.599 | 0.444 | -0.063 | 0.698 | 1.873 | 0.168 | NA | NA | NA |
| Undecanoic acid | 0.71±0.50 | 0.72±0.37 | 0.58±0.24 | <0.001 | 0.976 | -0.056 | 0.730 | 0.352 | 0.706 | NA | NA | NA |
| Lauric acid | 35.26±5.51 | 39.52±12.60 | 26.68±6.42 | 0.420 | 0.521 | 0.238 | 0.134 | 6.607 | **0.003**** | NS | **0.003**** | **0.020*** |
| Myristic acid | 176.87±35.39 | 209.83±75.23 | 141.38±31.23 | 0.383 | 0.540 | 0.289 | 0.067 | 4.972 | **0.012*** | NS | **0.009**** | NS |
| Pentadecanoic acid | 3.07±0.93 | 3.50±1.67 | 2.60±0.72 | 0.020 | 0.889 | 0.076 | 0.635 | 1.526 | 0.230 | NA | NA | NA |
| Palmitic acid | 2558.46±329.38 | 2765.48±609.57 | 1970.55±500.02 | 2.203 | 0.146 | 0.310 | **0.048*** | 8.287 | **0.001**** | NS | **0.001**** | **0.004**** |
| cis-10-Heptadecenoic acid | 3.16±1.10 | 3.88±1.48 | 3.01±0.77 | 0.005 | 0.947 | 0.110 | 0.495 | 1.634 | 0.209 | NA | NA | NA |
| Stearic acid | 2335.40±350.40 | 2617.71±623.07 | 1874.40±576.47 | 1.292 | 0.263 | 0.250 | 0.115 | 5.761 | **0.007**** | NS | **0.005**** | **0.045*** |
| Nonadecanoic acid | 8.82±3.38 | 8.71±3.39 | 6.06±1.95 | 2.751 | 0.105 | 0.258 | 0.104 | 2.660 | 0.083 | NA | NA | NA |
| Arachidic acid | 145.94±31.72 | 167.82±40.99 | 128.53±35.95 | 0.313 | 0.579 | 0.138 | 0.389 | 2.896 | 0.068 | NA | NA | NA |
| Behenic acid | 46.04±15.71 | 47.20±21.58 | 37.90±8.34 | 1.261 | 0.268 | 0.275 | 0.082 | 1.002 | 0.377 | NA | NA | NA |
| Lignoceric acid | 2.20±1.45 | 1.90±0.86 | 1.85±0.55 | 0.121 | 0.730 | 0.030 | 0.853 | 0.377 | 0.688 | NA | NA | NA |
| Oleic acid | 3.22±0.76 | 3.27±0.65 | 2.86±0.57 | 0.577 | 0.452 | 0.105 | 0.513 | 0.991 | 0.381 | NA | NA | NA |
| Linoleic acid | 3509.15±680.47 | 3107.77±1202.85 | 4906.00±1150.81 | 3.875 | 0.056 | -0.364 | **0.019*** | 10.014 | **<0.001***** | NS | **<0.001***** | **0.001**** |
| Linolenic acid | 1135.21±189.36 | 980.68±323.82 | 865.93±183.00 | 9.729 | **0.003**** | 0.479 | **0.002**** | 5.139 | **0.011*** | NS | NS | **0.011*** |
